# A Comprehensive Survey on Multi-Agent Reinforcement Learning for Connected and Automated Vehicles

**DOI:** 10.3390/s23104710

**Published:** 2023-05-12

**Authors:** Pamul Yadav, Ashutosh Mishra, Shiho Kim

**Affiliations:** School of Integrated Technology, Yonsei University, Incheon 21983, Republic of Korea

**Keywords:** autonomous vehicles, connected vehicles, multi-agent reinforcement learning, safety, unseen scenario

## Abstract

Connected and automated vehicles (CAVs) require multiple tasks in their seamless maneuverings. Some essential tasks that require simultaneous management and actions are motion planning, traffic prediction, traffic intersection management, etc. A few of them are complex in nature. Multi-agent reinforcement learning (MARL) can solve complex problems involving simultaneous controls. Recently, many researchers applied MARL in such applications. However, there is a lack of extensive surveys on the ongoing research to identify the current problems, proposed methods, and future research directions in MARL for CAVs. This paper provides a comprehensive survey on MARL for CAVs. A classification-based paper analysis is performed to identify the current developments and highlight the various existing research directions. Finally, the challenges in current works are discussed, and some potential areas are given for exploration to overcome those challenges. Future readers will benefit from this survey and can apply the ideas and findings in their research to solve complex problems.

## 1. Introduction

The recent success of deep reinforcement learning (DRL) in solving complex decision-making and control problems such as playing video games [1], locomotion in robots [2], and energy optimization in buildings [3] is due to the application of deep neural networks for function approximation to deal with tremendously large or continuous state–action pair spaces. Despite such developments in DRL algorithms, real-world utility lies in their application to scenarios with more than one agent, which is a typical case in the real world. Multi-agent reinforcement learning (MARL) is the application of reinforcement learning (RL) to a problem setting, where multiple agents make decisions in a common environment to optimize their long-term return through interactions with each other and the environment. Despite the various underlying challenges in its realization, there is massive interest in the research community to apply it to various exciting problems such as multi-player games such as Minecraft [4], Dota 2 [5], and soccer [6], unmanned aerial vehicles [7], autonomous vehicles [8,9], etc.

Among these, the autonomous vehicle (AV) is a highly complex and challenging area. Early research on autonomous vehicles achieved success around the 1990s with the development of the ALVINN project at Carnegie Mellon University [10]. This project demonstrated the capability of neural networks to perform lane-keeping tasks in AVs. Since then, initiatives such as the DARPA grand challenges have continued to push forward AV development [11] through various car manufacturers and tech companies, contributing to its commercialization to a certain extent [12]. This early effort has led to the development of advanced driver assistance systems (ADASs) such as adaptive cruise control (ACC), lane keeping assistance, and lane departure warning, which provide vehicles with partial autonomy and pave the way for full AVs. In parallel, information and communication technology (ICT) has brought advancement in transportation infrastructures, such as intelligent traffic management systems, traffic forecasting systems, intelligent traffic light control, etc., commonly categorized as intelligent transportation systems (ITSs) [13]. The advancement of CAVs heavily relies on the implementation of ITS technologies, so the discussion of autonomous vehicles (AVs) and intelligent transportation systems (ITSs) is grouped under the umbrella of connected and automated vehicle (CAV) development in this survey. First, the motivation for this survey originates from the fact that CAVs are rapidly advancing, and there is a growing interest in developing ITSs that can operate efficiently and safely in complex and dynamic environments. MARL is a promising approach to enable CAVs to learn from their interactions with other vehicles and agents in the environment and make decisions that optimize traffic flow, reduce congestion, and improve safety.

Second, there is a growing body of research on MARL for CAVs, with many different algorithms and techniques being developed. A comprehensive survey can help to synthesize this research and provide a clear overview of the state of the art (SOTA), including the strengths and weaknesses of different approaches. Third, many challenges are associated with applying MARL to CAVs, including scalability, coordination, communication, and safety. A comprehensive survey can help to identify these challenges and provide insights into how they can be addressed. Finally, there is a need for interdisciplinary collaboration between researchers in computer science, control theory, and transportation engineering to develop practical MARL algorithms for CAVs. A comprehensive survey can help to bridge these disciplines and foster new collaborations and ideas.

Many surveys exist on the topic of MARL, such as that by Hernandez-Leal et al. [14] who provided an overview of the recent works in MARL and classified them under four categories, i.e., emergent behaviors, learning communication, learning cooperation, and agents modeling agents. Du and Ding [15] reviewed MARL systematically, including classical algorithms, research progress, and practical applications. They also presented some future research directions in areas such as demonstration, model-free deep RL, and transfer learning for MARL. Nguyen et al. [16] surveyed the challenges in MARL and categorized them into five categories: non-stationarity, partial observability, multi-agent training schemes, multi-agent transfer learning, and continuous state and action spaces. Gronauer and Diepold [17] surveyed SOTA MARL papers and outlined various trends for future works. Wong et al. [18] surveyed the field of MARL from the challenge perspective and identified five significant challenges: centralized training and decentralized execution, opponent modeling, communication, efficient coordination, and reward shaping. Surveys on MARL for specialized domains also exist in the literature, such as vehicular networks [19] and future internet [20]. However, there is a lack of extensive surveys on autonomous vehicles in the current scenario. Schmidt et al. [21] surveyed the applications of MARL in autonomous mobility scenarios, but only scratched the surface of autonomous vehicles. To the best of our knowledge, the work by Dinneweth et al. [22] is the only survey in the current literature investigating the works specifically on MARL for autonomous vehicles. They have classified the works into four paradigms: mixed traffic, with or without socially desirable AVs, and fully autonomous traffic. Nonetheless, their survey focuses on a limited number of papers published between 2019 and 2022, suggesting the necessity for a more extensive examination of current research.

In this paper, we comprehensively survey the various CAV applications in the context of MARL by discussing their methodological (algorithmic) novelties. Additionally, we highlight the ongoing trends, current limitations, and future directions for researchers interested in this area. Our research contributions in this survey are highlighted below:

- This survey presents a comprehension of SOTA DRL techniques used in MARL.
- It discusses the uncertainty-aware algorithms used in motion planning to tackle the problems of a real-time environment.
- It includes the learning paradigms and techniques of MARL with their details.
- It describes the open-source simulators available for CAV applications.
- Useful datasets available in the public domain have also been introduced in this survey.
- It incorporates the popular applications of CAVs and involved techniques with their advantages and limitations.
- Finally, it presents the shortcomings and research gaps in the CAV domain and suggests future directions to fill this gap using MARL techniques.

This comprehensive information benefits future readers in tackling their research problems in the CAV domain.

The rest of the paper is organized as follows. Section 2 discusses the mathematical formulation of MARL under different learning schemes. Section 3 presents a detailed survey of the recent papers on MARL for CAV. Section 4 discusses the trends, challenges, and potential research directions. Section 5 concludes the paper by summarizing the findings and future directions.

## 2. Multi-Agent Reinforcement Learning

**Figure 1:**
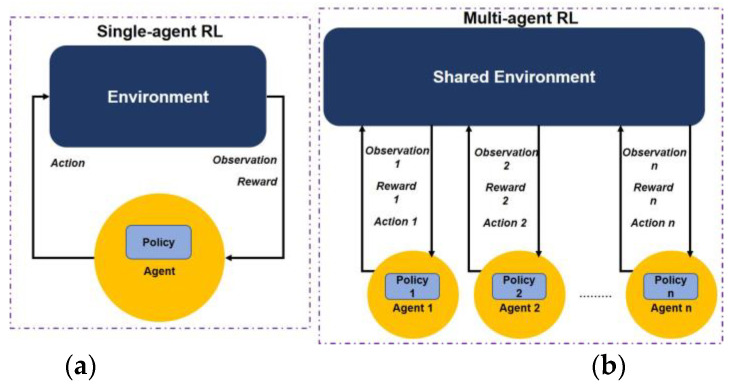
A diagrammatic representation illustrating the difference between (**a**) single-agent RL framework and (**b**) multi-agent RL framework.

Reinforcement learning (RL) is generally used to train an agent’s policy to learn to act optimally in its environment. Figure 1 illustrates a conceptual difference between single-agent RL and multi-agent RL frameworks. The agent can take actions based on the observations (or states) it receives from the environment, and for every change in the state, it may (or may not) receive a reward. RL is traditionally a sequential decision-making problem and is formalized by a Markov decision process (MDP). The MDP is defined as a tuple:

(1) $$\left(S,\;A,\;P,\;R,\gamma \right)$$

where S, A are state and action space, respectively.

(2) $$P:S\times A\to P\left(S\right)$$

P is defined as the transition probability function for each state, where the state can be either known or unknown for a given problem.

(3) R:S×A×S→R

R is the reward function which is a scalar value obtained by the agent from the environment on making a state transition and γ is the discount factor such that $\gamma \in \left[0,1\right)$. Figure 1 shows an RL framework for a single agent interacting with its environment, where $o_{t}$ represents the observation (or state information) received and $a_{t}$ represents the action taken by the agent at time step t and $r_{t+1}$ represents the reward signal received at the next time step t+1. A classical work by Sutton and Barto provides an in-depth discussion of RL [23].

In contrast to single-agent RL, multi-agent RL assumes the existence of more than one agent interacting within a shared environment. Multi-agent RL problems are formalized using Markov games [24], an extension of MDPs to multiple agents. In general, a Markov game (MG) is a tuple:

(4) $$\left(N,S,\left\{A^{i}\right\},\;P,\left\{R^{i}\right\},\gamma \right)$$

where N is the number of agents, S is the state space consisting of the states observed by all the agents, $\left\{A^{i}\right\}$ is the action space of the ith agent such that $\left\{A^{1}\right\}\times \ldots \times \left\{A^{N}\right\}=A$ is the joint action space of all the agents. P (defined by (2)) represents a transition probability function for each state, which can be either known or unknown for a given problem, $R^{i}$ (defined by (3)) is the reward function, which is a scalar value obtained by the ith agent on making a state transition, and γ is the discount factor such that $\gamma \in \left[0,1\right)$. The goal is to maximize the discounted sum of rewards over a given episode. A value function $V_{\pi^{i},\pi^{i^{\prime }}}^{i\left(s\right)}\;$ an agent i in a Markov game is defined as follows:

(5) $$V_{\pi^{i},\pi^{i^{\prime }}}^{i\left(s\right)}=E_{s_{t+1}\sim \;P,\;\;a_{t}\;\sim \;\pi }{[\sum }_{t=0}^{\infty }\gamma^{t}R^{i}\left(s_{t},a_{t},s_{t+1}\right)|\;s_{0}=s]$$

It evaluates the goodness of a joint policy π for the ith agent. π can be written as a collection of an individual agent’s policies as:

(6) $$\pi =\left\{\pi^{1},\pi^{2},\ldots \;,\;\pi^{n}\right\},$$

and

(7) $$\pi^{i^{\prime }}=\pi^{1},\pi^{2},\ldots \;,\;\pi^{i-1},\;\pi^{i+1},\;\ldots ,\pi^{n}\}$$

A Nash equilibrium solution can be defined for a Markov game when each agent’s optimal policy is $\pi_{*}^{i}$ and the following inequality holds:

(8) $$V_{\pi_{*}^{i},\pi_{*}^{i^{\prime }}}^{i\left(s\right)}\geq \;V_{\pi^{i},\pi_{*}^{i^{\prime }}}^{i\left(s\right)}\forall s\in S,\;\forall \;i\;\pi^{i}\in \Pi^{i}$$

A multi-agent reinforcement learning problem can be defined as belonging to one of the following three scenarios depending on the nature of interaction among the agents: cooperative, competitive, and mixed [18]. In a fully cooperative scenario, the agents are expected to collaborate with each other to solve a common task and aim to maximize a common return jointly for all the agents. In a fully competitive scenario, the agents compete for a zero-sum game, where only one agent may emerge victorious, therefore, the agents are expected to maximize their individual rewards while trying to minimize other agent’s rewards. Some examples of competitive scenarios are car racing, blackjack, chess, etc. In a mixed scenario, the aim is to maintain a balance between collaborating and competing with each other. Some examples are soccer, basketball, and other team games.

**Figure 2:**
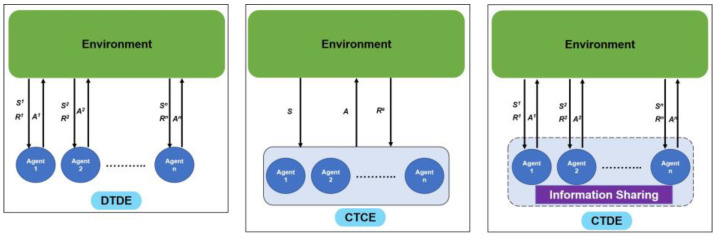
Schematic diagram of the three popular learning paradigms in multi-agent RL setting.

It is essential to note that CAVs in real-world settings inherently assume a cooperative scenario where each vehicle (hereafter, “vehicle” is synonymous with “agent”) is expected to cooperate while maximizing its rewards. Depending on the nature of training and execution, cooperative multi-agent RL algorithms are classified into three learning paradigms. Figure 2 shows a pictorial representation of the three learning paradigms.

### 2.1. Decentralized Training and Decentralized Execution (DTDE)

In this paradigm, each agent i has its policy such that it maps its local observations to individual action distribution denoted as $\pi^{i}:S^{i}\to P\left(A^{i}\right)$. Additionally, it does not share information as each agent learns its policy independently. One disadvantage of not sharing the information is that it leads to non-stationarity in the environment, exacerbating the learning process. Researchers have found that distributed learning methods perform relatively less well than centralized training paradigms [25]. Despite these limitations, DTDE paradigms have been widely applied to solve tasks such as cooperative navigation [26]. Some recent works have successfully applied the DTDE paradigm by utilizing a decentralized experience replay called concurrent experience replay trajectories (CERTs) to allow independent learners to stabilize the sample generation [27]. Another work improved the independent learners’ ability by introducing an additional reward approximation and a prioritized replay strategy.

### 2.2. Centralized Training and Centralized Execution (CTCE)

The CTCE paradigm uses a centralized learner whose goal is to learn a joint policy for all the agents denoted as $\pi :S\to P\left(A\right)$. This joint policy maps the distributed observations to a set of distributions for individual actions. One condition in the case of CTCE is that there should be unhindered and instant communication between the agents. Generally, single-agent RL algorithms such as DDPG [28] or actor–critic [29] are applied directly to the multi-agent problems in this paradigm. In the scenario having a relatively more significant number of agents, learning in the CTCE paradigm may suffer from the curse of dimensionality as the number of total state–action spaces for all the agents could grow exponentially, leading to an intractable situation for finding an optimal joint policy. One common technique for reducing the effects of high dimensionality is to partition the joint policy into the individual policies of each agent and train them separately while enabling them to exchange information with one another [25]. Mathematically, this can be expressed as $P\left(A\right)=\Pi_{i}P\left(A^{i}\right)$. However, this leads to another issue called the “lazy agent problem”, where one agent may have a smaller incentive to learn a good policy, as its actions may prevent another agent from learning a better policy, leading to an overall lower reward. In the lazy agent problem, the agents in a team are not performing equally well, but they are still receiving the same collective reward. To address this issue, researchers have suggested various learning and non-learning methods that assign credit to each agent based on their individual contributions [30,31,32,33,34,35]. Interestingly, the centralized training and decentralized execution indigenously have no issues or occurrences of lazy agents.

### 2.3. Centralized Training and Decentralized Execution (CTDE)

Both CTCE and DTDE have their share of disadvantages, due to which a hybrid paradigm is utilized to design modern MARL algorithms. In this paradigm, each agent has its policy such that it maps its local observations to individual action distribution $\pi^{i}:S^{i}\to P\left(A^{i}\right)$. One key difference from CTCE is that the additional information supplied during the training phase is discarded during test time. During the training phase, agents can increase their overall learning speed and overcome non-stationarity in the environment by sharing resources such as computing power and learned knowledge. Mutual information enables agents to associate the outcomes of actions with their corresponding agents, which facilitates sharing resources such as computing power and learned knowledge during the training phase in CTCE.

## 3. MARL for CAVs

**Figure 3:**
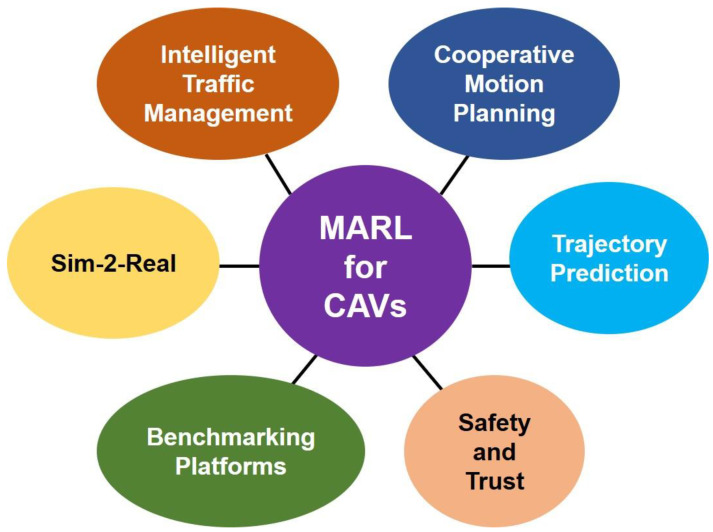
A taxonomy of categories of MARL for CAVs.

A taxonomy of typical MARL applications in CAVs is exhibited in Figure 3. One of the primary objectives of this survey is to identify the ongoing research directions in MARL for the CAV domain. This section briefly reviews the representative papers, particularly mentioning the problems tackled in each work and the proposed solutions.

Any given work may be suited to more than one category, but to maintain the paper’s clarity, it is mentioned only under one category in the following subsections. Table 1 provides a tabular analysis of the surveyed papers, which may contain the classification of any paper belonging to more than one category. Through the survey, the papers are categorized as follows: application areas, sim-to-real approaches, safety, and benchmarking platforms. A summary of popularly used simulators is provided before detailing the paper analysis.

### 3.1. Simulators

**Figure 4:**
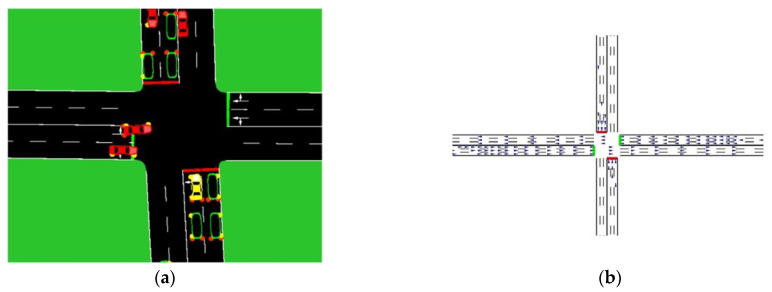

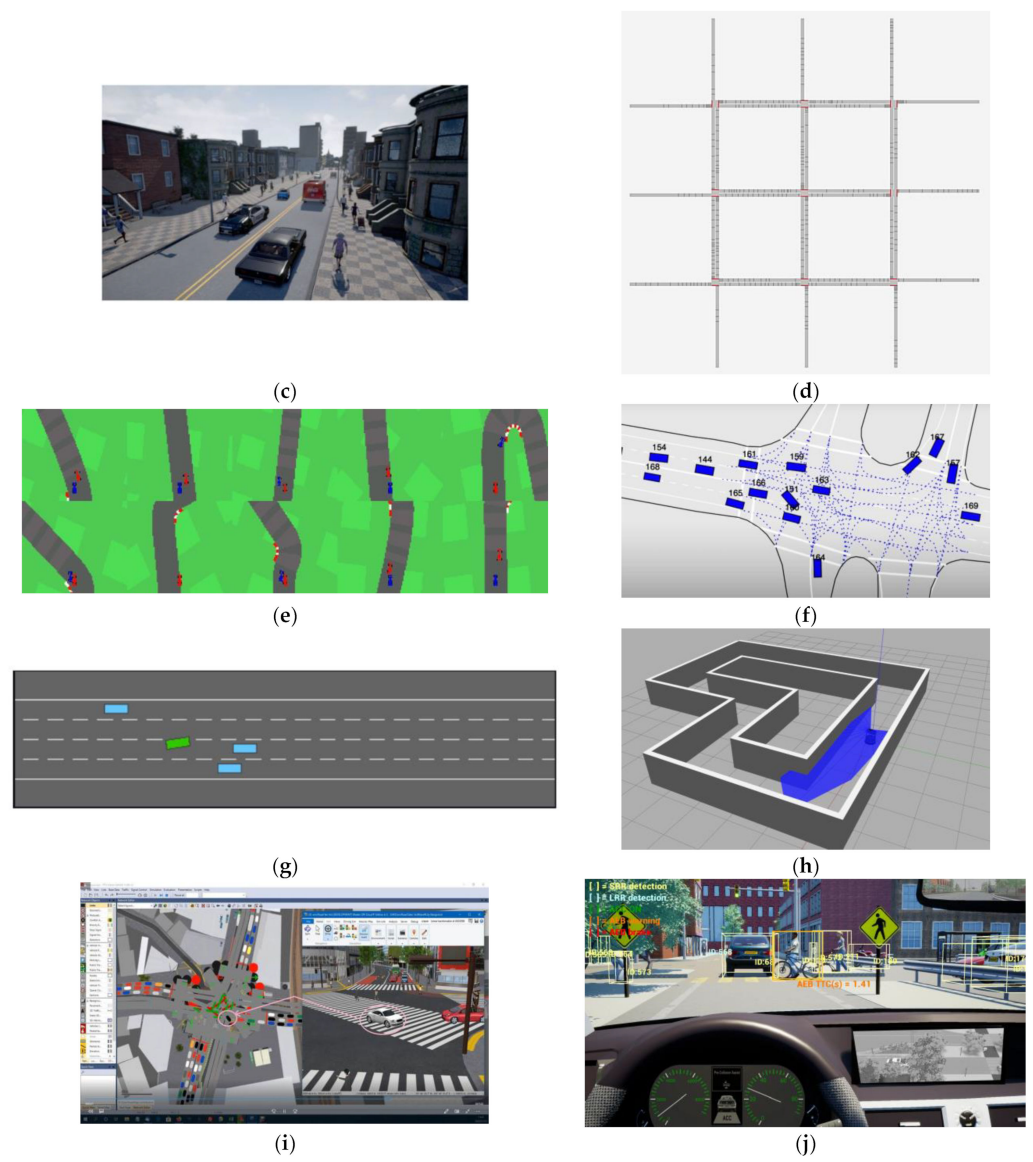
Popular CAV simulators: (**a**) SUMO; (**b**) CityFlow; (**c**) CARLA; (**d**) OpenAI traffic simulator; (**e**) Multi-Car Racing Gym Environment; (**f**) INTERSECTION data visualization tool; (**g**) Highway-env; (**h**) Gym-Gazebo; (**i**) PTV VISSIM; and (**j**) PRESCAN.

CAV simulators provide a fruitful method to design autonomous vehicle scenarios under varying conditions and evaluate the performance of algorithms. Figure 4 illustrates popular simulators used for CAVs.

Some of the popular open-source and proprietary simulators and frameworks used in the surveyed papers are:

CityFlow [36]: A MARL environment for large-scale city traffic scenario testing. Based on synthetic and real-world data, it can simulate road networks and traffic flow. It allows users to simulate and analyze various traffic scenarios in real time, including intersections, roads, and highways. The simulator incorporates a variety of traffic models, including car-following, lane-changing, and decision-making models, to accurately simulate the behavior of different types of vehicles, such as cars, buses, and bicycles. CityFlow is designed to be modular and extensible, enabling users to easily customize and extend the simulator to meet their specific needs. It also provides various visualization tools for users to view the simulation results in real time, including traffic flow, speed, and congestion levels.

Simulation of Urban Mobility (SUMO [37]): A popular open-source traffic simulation platform that models and simulates transportation systems. It allows users to simulate traffic scenarios on different scales, from individual vehicles to large-scale road networks. Similar to CityFlow, it also allows users to simulate and analyze various traffic scenarios in real time, including intersections, roads, and highways. Additionally, SUMO can be easily integrated with other traffic management and control systems to test and validate their performance in simulated environments.

Flow [38]: A framework for benchmarking traffic control, offering a collection of traffic control scenarios, tools for creating custom scenarios, and integration with deep reinforcement learning and traffic microsimulation libraries, and used together with SUMO for designing the vehicle simulations.

CARLA: An open-source autonomous driving simulator designed to be modular and flexible to serve a wide range of tasks in autonomous driving research and development [39]. It is designed to provide a platform for researchers and developers to test and evaluate algorithms for autonomous vehicles in a virtual environment replicating real-world conditions. CARLA offers a realistic 3D world with dynamic and complex scenarios that allow testing and training of various autonomous driving tasks such as perception, motion planning, control, and coordination. It simulates sensors such as cameras, LIDAR, and RADAR and provides a range of prebuilt scenarios and customizable parameters.

Highway-env: An open-source Python-based simulator for developing and evaluating algorithms for autonomous driving on highways. It is designed to be simple, modular, and easy to use, emphasizing providing a customizable platform for researchers and developers to experiment with different autonomous driving scenarios and evaluate their algorithms. It is a minimalist environment capable of simulating various traffic scenarios such as highway driving, lane merging, roundabouts, parking, intersections, racetracks, etc. [40].

Multi-Car Racing Gym Environment: An open-source reinforcement learning environment designed for testing and developing autonomous driving agents for multi-agent racing scenarios. It is built on top of the OpenAI gym framework, making it easy for researchers and developers to use with existing reinforcement learning libraries such as TensorFlow and PyTorch. The environment simulates a multi-agent racing game where each agent controls a vehicle that competes against other agents on a racetrack. The agents can take actions such as accelerating, braking, and steering to navigate the track and try to finish the race in the shortest time possible. The environment provides different types of tracks with varying difficulty levels, making it suitable for testing and training a wide range of autonomous driving algorithms [41].

INTERACTION Visualization Tool: A Python-based tool for visualizing the traffic scenarios provided in the INTERACTION dataset [42]. It provides a minimalistic visualization environment that simulates the various traffic scenarios defined in the INTERACTION dataset.

OpenAI Traffic Simulator: A gym-based traffic simulator for designing and simulating a grid-based traffic intersection [43]. It provides a minimalistic environment capable of simulating simple cross-lane traffic intersections of single lanes in each direction.

Gym-Gazebo: Another open-source toolkit that integrates the Gazebo robot simulator with the OpenAI Gym reinforcement learning framework. It provides a platform for researchers and developers to train and test their reinforcement learning algorithms in a realistic, physics-based simulation environment. Gazebo is a popular robot simulator that can simulate complex physics, sensor models, and dynamic environments. Gym-Gazebo provides an interface between Gazebo and OpenAI Gym, allowing for easy integration with existing reinforcement learning libraries and algorithms. With Gym-Gazebo, researchers can simulate various robotic tasks, such as navigation, manipulation, and vision-based tasks. The environment provides a range of sensors, including camera, LIDAR, and depth sensors, which can train agents to perceive the environment and make decisions based on the data collected [44].

PTV VISSIM: VISSIM is a microscopic traffic simulation software developed by PTV Group [45]. It is used to model, simulate, and analyze traffic flow and behavior of vehicles, pedestrians, bicycles, and public transport in a virtual environment. VISSIM can simulate a wide range of transportation scenarios, including intersections, roundabouts, highways, urban streets, and public transportation systems. It is commonly used by transportation planners, engineers, and researchers to evaluate and optimize the design and operation of transportation systems.

PRESCAN: A simulation software for the development and testing of advanced driver assistance systems (ADASs) and autonomous driving (AD) systems [46]. It is developed by TASS International, a company now part of Siemens Digital Industries Software. PRESCAN allows users to create virtual models of vehicles and their surroundings, including roads, intersections, pedestrians, and other vehicles. The software simulates various scenarios and environmental conditions, such as different weather conditions, lighting, and road surfaces, to test the performance and safety of ADASs and AD systems. 

**Table 1:** Summary of the surveyed papers highlighting their representative work in the MARL and CAV domain.

Reference No.	Category	Algorithmic Contributions	Test Scenario	Simulator/Framework
[47]		Policy gradient without Markovian assumptions, option graph with a gating mechanism	Double merge scenario	Custom
[48]	Cooperative motion planning	LSTM ^#^, REINFORCE	Vehicle platooning on freeway	Highway-env simulator
[49]		Value function-based RL, kinematics constraint encoding	Two-agent collision avoidance	Custom
[50]		Q-learning, graph convolution network	On-ramp-merging scenarios	SUMO
[51]		Coordination graphs, max-plus algorithm	Lane-free driving	Flow, SUMO
[52]		Curriculum learning, PPO ^$^	Stop-and-go wave	SUMO, Veins
[53]		Hierarchical MARL	One-to-One racing	Kart racing environment
[54]		Multi-agent advantage actor–critic, parameter sharing	Lane-changing scenario	Highway-env simulator
[55]		Mean field multi-agent DQN ^%^	Dynamic routing problem	SUMO
[56]		Curriculum learning, PPO	Bidirectional driving on a narrow road	Multi-Car Racing Gym Environment
[57]		Shapley value-based reward allocation	Lane-changing scenario	CARLA
[58]		Altruism as convex optimization	Highway merging	Custom
[59]	Trajectory prediction	Latent representation learning for RL	Lane-merging scenarios	CARLA
[60]		Continual learning, graph neural network	INTERACTION dataset scenarios	INTERACTION dataset visualization tool
[61]		Graph neural network, ego- and allocentric approach	INTERACTION and TrajNet++ dataset scenarios	INTERACTION dataset visualization tool
[62]		Graph attention network, parameter sharing	INTERACTION and NGSIM dataset scenarios	INTERACTION dataset visualization tool
[63]		Spatiotemporal graph autoencoder, kernel density estimation	MAAD dataset	Multi-Car Racing Gym Environment
[64]	Intelligent traffic management	Curriculum learning, LSTM	Unsignalized intersection	SUMO
[65]		Fastest crossing time point algorithm, MA-DQN	Unsignalized intersection	Custom (Python-based simulation)
[66]		Delay aware Markov game, MA-DDPG ^£^	Unsignalized intersection	Highway-env simulator
[67]		Outflow congestion (new metric), transfer RL	Traffic congestion	SUMO
[68]		Game-theoretic auction mechanism	Unsignalized intersection, roundabout, merging scenarios	OpenAI traffic simulator
[69]		Transfer planning, max-plus coordination, DQN	Unsignalized intersection	SUMO
[70]		Independent Q-learning	Cooperative traffic light control	VISSIM
[71]		Multi-agent advantage actor-critic, multiple local learning agents, spatial discount factor	Cooperative traffic light control	SUMO
[72]		Graph neural network, recurrent neural network	Cooperative traffic light control	CityFlow
[73]		Nearest-neighbor-based state representation, pheromone-based regional green-wave	Cooperative traffic light control	SUMO
[74]		Contextual DQN, contextual actor–critic	Cooperative fleet management	Custom
[75]	Sim-to-real	LSTM, epistemic, and aleatoric uncertainty estimation, MPC	Optimal parking assignment, NGSIM, HighD, and INTERACTION dataset	INTERACTION dataset visualization tool
[76]		QMIX	Optimal parking assignment	Custom
[77]	Sim-to-real/Safety	Lyapunov function, soft actor–critic	Obstacle avoidance while driving	Gym-Gazebo
[78]	Safety	DDPG	Distracted pedestrian avoidance	PVI framework
[79]		TD3 ^§^, MPC ^€^	Crash avoidance, lane keeping	Custom
[80]	Safety	Shielding, CQL ^¤^, MADDPG	Lane-free traffic control	SUMO
[81]	Benchmarking platform	MCTS ^¥^, RL-based models	Ego-vehicle velocity control	BARK
[82]		Unified approach	Multiple scenarios (ring, highway ramp, etc.)	SUMO
[83]		Partially observable Markov game	Unsignalized intersection	MACAD-Gym
[84]		Model-based offline RL for trajectory planning	NGSIM dataset scenarios, unprotected left turn at the intersection	NGSIM, CARLA

^#^ LSTM- Long Short-Term Memory; ^$^ PPO—Proximal Policy Optimization; ^%^ DQN—Deep Q-Network; ^£^ DDPG—Deep Deterministic Policy Gradient; ^§^ TD3—Twin Delayed DDPG; ^€^ MPC—Model Predictive Control; ^¤^ Conservative Q-Learning; ^¥^ MCTS—Monte Carlo Tree Search.

### 3.2. Application Areas

#### 3.2.1. Cooperative Motion Planning

Cooperative motion planning is an emerging area focused on a fully cooperative CAV environment. Cooperative adaptive cruise control (CACC) is one of the primary systems developed to deal with motion planning that assists in the autonomous maneuvering of vehicles. CAVs share crucial information, such as velocity, acceleration, braking conditions, etc., among each other for cooperative motion planning. The efficiency of the cooperative adaptive cruise control (CACC) system depends on the quality of the vehicle-to-vehicle (V2V) communication between the connected vehicles. CACC provides a variety of features, including vehicle platooning for optimal speed and lane-changing actions and automation of complex highway merging tasks. These capabilities enable smooth driving maneuvers, which result in a more pleasant ride for passengers in fully autonomous vehicles [85].

Shalev-Shwartz et al. [47] applied the DRL approach to a long-term driving strategy problem under a multi-agent scenario. The authors minimized the gradient estimation variance in this work using policy gradient iterations without Markovian assumptions. They decomposed the problem into two components: desires and hard constraints. The policy composition for desires aims to enable comfortable driving, whereas hard constraints ensure safety. In addition, they introduced an option graph consisting of a gating mechanism to diminish the effective horizon and variance of gradient estimation. The option graph helps in reducing the overall sample complexity. The proposed method was tested for a simulated driving scenario of double merging, where vehicles approach a merge lane from two sides, choose whether to merge or not, and show a reasonable performance for the task.

Peake et al. [48] developed a platooning-based CACC system to coordinate the driving of vehicles in a CAV network. An LSTM model was used to design the ACC controller of each agent such that n-simultaneous LSTM blocks were combined to create a chain of ACC controllers, forming a unified CACC system. A policy gradient-based RL algorithm called REINFORCE was used to train each agent’s policy. Cooperatively, the unified policies formed a communication protocol that was used to stabilize the driving of the whole platoon. This system was tested under a stop-and-go scenario for a platoon of three to five cars on a simulated freeway. Various metrics, such as rear-end collisions, string stability, and traffic jams, were used to test its effectiveness. This model works well even when complete, stable communication among the platoon vehicles is not guaranteed.

Chen et al. [49] tackled the problem of multi-agent navigation via cooperative collision avoidance in a decentralized non-communicating environment. In this work, the authors considered a two-agent collision avoidance scenario and utilized a value-based function to optimize each agent’s learning policy. The physical avoidance between the agents is encoded using each agent’s kinematic constraints. The performance of each agent is evaluated through the time it takes to reach the goal while encountering another agent and avoiding it in its trajectory. This work has shown an improvement in path quality (i.e., time to reach a goal while avoiding collision) by 26% over the SOTA technique.

Chen et al. [50] considered local and global driving information important factors in developing a CACC system. Local information is obtained via cooperative sensing through in-built sensors such as cameras and LIDARs, whereas global information is obtained through V2V communication among the nearby vehicles. The CAV network is a graph incorporating topological information about the traffic environment. In addition, a Q-learning algorithm is fused with a graph convolutional network (GCN) to propose a novel GCQ model to perform various tasks, such as lane change and highway merging. This model is demonstrated to be capable of performing zero-shot learning under different scenarios such as dynamic traffic densities.

Troullinos et al. [51] tackled the problem of CAV control in a lane-free environment. The local interaction of the vehicles was modeled using coordination graphs [86], combined with the max-plus algorithm [87] to maximize the agents’ joint action. Artificial potential fields [88] were used to construct local utility functions that assist collision avoidance and vehicle velocity optimization. The experiments were performed using the Flow framework with the SUMO simulator.

Li et al. [52] considered the problem of fuel consumption optimization under an autonomous vehicle platooning ecosystem. They proposed a curriculum learning-based multi-agent RL framework to accomplish this goal by optimally controlling the vehicle’s platoon velocity to maintain its safety and efficiency. To this end, a novel communication protocol consists of a state-transmission channel for transmitting the appropriate state information to handle the partial observability and a reward-transmission protocol that assigns explicit rewards to each agent to deal with the “lazy agent” problem. 

Thakkar et al. [53] considered the problem of multi-agent planning in a racing car environment where the goal is to win the race while performing collision avoidance and lane changing. They developed a hierarchical controller capable of learning a high-level policy for planning the racing tactics and a low-level one for motion planning of the racing cars. Their algorithm was tested on a kart racing environment for different racing tracks, e.g., oval-shaped and random-curve tracks. The algorithm was demonstrated to outperform the baseline algorithms in terms of total wins and safety scores.

Zhou et al. [54] presented a decentralized cooperative multi-agent reinforcement learning (MARL) approach for autonomous vehicles to perform lane-changing tasks in mixed traffic. The authors introduced a new multi-agent advantage actor–critic network model that is efficient and scalable and uses a parameter-sharing mechanism to communicate the learned knowledge. The performance of this model was evaluated through a comprehensive empirical analysis involving three different traffic densities and two levels of human driver behavior. The results show that the model leads to improved driving safety, efficiency, and passenger comfort compared to SOTA models.

Shou et al. [55] considered the approach of the Markov routing game to model the interaction between adaptive agents and traffic congestion. The proposed approach allows the agent to perform repeated interactions with other agents and the traffic environment to learn optimal policies even when they have limited or incomplete information about the environment. A MARL algorithm was developed that uses a mean action, representing the traffic flow on a chosen link after an agent enters it, to capture the competition among agents. This approach is particularly efficient for large problems with many agents.

Şehriyaroğlu and Genç [56] proposed a new scenario where the vehicles drive in opposite directions on a narrow bidirectional single-lane road without any form of communication among them. In this scenario, the goal was to pass other vehicles without colliding with them, which was accomplished via a new reward function designed to approximate the problem. In this work, it is also demonstrated that curriculum learning reduced the training time for the agents.

Han et al. [57] proposed a novel method for reward reallocation in a CAV environment. This method utilizes Shapley values [89] to reallocate the total system’s reward among the agents, which motivates more cooperation and information sharing among the CAVs in the network. The authors developed an actor–critic-based algorithm with the proposed reward mechanism to train a cooperative policy for lane-changing tasks. The algorithm was tested on agents in a CARLA environment under both fully automated and mixed traffic scenarios. It was demonstrated to outperform popular MARL algorithms such as MADDPG [90], M3DDPG [91], and COMA [92].

Toghi et al. [58] proposed a MARL framework for training altruistic vehicle agents that can navigate safely on highways and cooperate in a mixed traffic scenario involving human drivers. They developed a policy dissemination algorithm allowing agents to learn from experience without needing an explicitly defined human driver behavioral model. The simulation showed that an optimal value for the level of altruism could be obtained, leading to improved traffic safety and flow under a highway merging scenario.

There is a wide range of tasks for the smooth maneuvering of CAVs, such as vehicle platooning, lane changing, lane merging, etc. As aforementioned, researchers used value function-based RL methods [49,57] to achieve these tasks. However, such methods fail to accommodate a large number of vehicles. The overall action space may grow exponentially, leading to an intractable situation of computing action value for each state–action pair. To overcome this shortcoming, some researchers have utilized policy-based RL methods [47,48,52,56] such as REINFORCE [48], DDPG, or actor–critic methods such as PPO [52,56], etc. Such methods can discover an optimal policy without the explicit need to compute the action value function for each state–action pair. LSTMs are frequently used to model the temporal correlation among the CAVs in a platoon [48]. These methods may succeed for a small number of vehicles, however, in a large CAV platoon, LSTMs may be slower to learn the correlation online due to their long sequential architecture. In addition, they are not capable of learning spatial correlation among the CAVs. Some researchers have considered GCNs to capture both the spatial and spatiotemporal correlation among the CAVs. However, they consume more computational resources and take longer to train. Transformers have already been demonstrated to perform better than CNNs for machine vision tasks, whereas LSTMs perform better for sequential data-based tasks. Therefore, the use of attention mechanism-based methods such as Transformers may be one of the possible candidates to solve the issue of GCNs’ complexity. 

#### 3.2.2. Trajectory Prediction

Trajectory prediction plays a key role in the safety enhancement of CAVs in a multi-agent setting. A robust and accurate trajectory prediction system should be able to predict the trajectories of other entities such as vehicles (CAVs and human-driven vehicles (HDVs)), pedestrians, etc. Such a system allows an autonomous vehicle to cope with unexpected changes in the traffic environment, such as sudden braking of nearby vehicles, sudden lane changing, etc. [93].

Xie et al. [59] proposed an approach to model the real-time dynamic hidden intentions of the surrounding agents in a mixed multi-agent setting. Their algorithm leverages latent representations of nearby agents’ policies to handle the multi-agent interaction scenario under a non-stationary environment. The study employs two experimental scenarios, namely 2D and 3D driving (CARLA), to show that the agent can effectively learn other agents’ hidden intentions while driving, which could be valuable in addressing trajectory prediction challenges.

Ma et al. [60] proposed a graph neural network-based trajectory prediction algorithm. Continual learning problems inspire their work, as they have developed a conditional generative memory model and episodic memory buffer to reduce catastrophic forgetting in the network. They tested their algorithm on several INTERACTION dataset traffic scenarios and showed SOTA performance.

Jia et al. [61] highlighted the possibility of bias in multi-agent trajectory projection problems when they are reformulated as a group of single-agent trajectory prediction problems. The authors proposed an efficient GNN-based multi-agent joint prediction model, treating all agents symmetrically in the graph and decoupling node and edge feature updates for improved efficiency. The “Node update” step collects and summarizes other agents’ information in an egocentric view. The “Edge update” step collects information in allocentric views from each neighbor, resulting in a combined egocentric-and-allocentric view that encourages shared parameters to learn underlying interactions among agents. This model achieved SOTA performance on the INTERACTION [42] and TrajNet++ [94] datasets and also increased running speed by 1.7–4.5 times compared to common multi-inference single-prediction models.

Mo et al. [62] focused their work on the future trajectory prediction of multiple agents in urban and highway driving scenarios using individual dynamics, interactions, and road structure. They introduced a novel heterogeneous edge-enhanced graph attention network (HEAT) to model and learn the interactions among the agents. It consists of a gate-based map selector that allows selective sharing of map information across all target agents instead of storing a localized map for each agent or sharing the same map across all agents. They validated the method on urban (INTERACTION) and highway (NGSIM) [95] driving datasets, showing that the proposed method can simultaneously predict multi-agent trajectories of an arbitrary number of heterogeneous agents while attaining SOTA performance.

Wiederer et al. [63] presented a model called the spatiotemporal graph autoencoder (STGAE) to perform anomaly detection in multi-agent trajectory prediction. It performs trajectory embedding and learns a latent representation of multi-agent trajectories, simultaneously learning multiple trajectories for a dynamic number of agents. Anomaly detection is carried out by performing kernel density estimation [96] on the latent representation of the STGAE, capturing the distribution of the normal trajectory data, and detecting anomalies in low-density regions of the estimated density. The authors also proposed the MAAD dataset for multi-agent anomaly detection using the OpenAI Gym MultiCarRacing-v0 environment, adapted for simulating anomalies on a two-lane highway shared by two vehicles controlled by human players.

The trajectory prediction aims to perform smooth and collision-free maneuvering and prevent traffic congestion under a fully CAV or mixed vehicle environment. As mentioned above, researchers have employed graph neural network (GNN)-based methods to accomplish these tasks [60,61,62,63,94,95]. GNNs are well suited for modeling inter-vehicular relations, such as spatiotemporal relations among the CAVs. Despite their utility, they may suffer from catastrophic forgetting as the network grows (i.e., it incorporates more vehicles). To overcome this issue, some researchers have utilized continual learning-based approaches [60]. Learning latent representations using autoencoders is yet another approach employed in this area and has been demonstrated to perform extremely well. 

#### 3.2.3. Intelligent Traffic Management (ITM)

Developing an intelligent transportation system relies heavily on efficient and adaptive traffic management. Some primary challenges in this category are automated intersection management, intelligent traffic light control, and dynamic traffic forecasting.

Guillen-Perez and Maria-Dolores [64] have considered the problem of automated intersection management. AIM algorithms are generally created using simple control algorithms incapable of learning or adapting to new situations. A new advanced AIM approach called advanced reinforced AIM (adv. RAIM) is presented, based on end-to-end multi-agent deep reinforcement learning (MADRL) and trained using curriculum through self-play. Adv. RAIM consists of a recurrent module (LSTM) to address the problem of variations as a function of some vehicles. LSTM allows the model to capture long-term spatiotemporal dynamics of the traffic network and help perform path planning.

Xu et al. [65] have attempted to achieve cooperative control of multiple non-signalized intersections using a vehicle–road collaboration-based architecture. The proposed work provides precise crossing decisions for vehicles using a fastest crossing time point (FCTP) algorithm, and for cooperative management of multiple unsignalized intersections, a multi-agent-based deep reinforcement learning scheduling (MADRLS) algorithm is used. Simulation results show that their approach outperforms traditional traffic light-based intersection management in terms of intersection throughput and vehicle waiting time.

Chen et al. [66] considered the problem of information delay in autonomous driving systems, such as communication delays, sensor delays, decision-making times, and actuator delays in the powertrain and hydraulic brake systems. To tackle this challenge, they proposed a general model for multi-agent systems with delays, called the delay-aware Markov game (DAMG), which extends the standard Markov game to include agent delays and tests the model for an unsignalized intersection scenario. Additionally, they presented a delay-aware training algorithm for DAMGs that combines centralized training and decentralized execution to stabilize multi-agent training.

Cui et al. [67] have proposed a new metric to alleviate the issue of traffic congestion under mixed-traffic settings. The proposed metric, outflow congestion, measures the traffic efficiency in open networks, and this metric is much more advantageous than the average-speed metric and is resistant to manipulation. The paper introduces two reinforcement learning-based methods, namely the modular approach and the transfer reinforcement learning approach, to tackle the intricacy of simulated scenarios involving hundreds of vehicles. These methods demonstrate superior performance compared to human-driven traffic. Moreover, it is among the first studies to demonstrate that a fully distributed multi-agent driving policy, which does not necessitate communication, can effectively alleviate congestion in an open road network scenario.

Chandra and Manocha [68] highlight the lack of guarantee of collision-free or deadlock-free navigation at unsignalized intersections, roundabouts, and merging scenarios. They have proposed a game-theoretic optimal auction methodology called GAMEPLAN, in which vehicle trajectories and velocities estimate a driver’s aggressiveness or impatience. GAMEPLAN uses driver behavior to define the bidding strategy and outputs an optimal turn-based ordering in which more aggressive or impatient drivers move first. GAMEPLAN is shown to be superior to other multi-agent planning methods in terms of reducing collisions and deadlocks in unsignalized intersections, roundabouts, and merging scenarios.

Van der Pol and Oliehoek [69] explored the problem of traffic light control using deep RL to coordinate multiple intersections. The authors used transfer planning [97] and the max-plus coordination algorithm [87] with the DQN algorithm to tackle this problem. They demonstrated their work in simulated traffic signal control in a SUMO environment. It is one of the earlier works which compares performance against various single-agent reinforcement algorithms.

Prabuchandran et al. [70] have tackled the problem of traffic signal control by re-formulating it as a multi-agent coordination problem. The authors utilized an independent Q-learning-based method to optimize each agent’s (traffic intersection) actions. The proposed method tackles the curse of dimensionality by localizing the control of each agent. Each agent’s Q-values are updated either using an epsilon-greedy or UCB-based exploration. The proposed method has been tested on two different traffic settings of road networks: a nine-junction road network and a twenty-junction road network, both modeled after the real road networks in Bangalore, India, and the simulation was performed using a VISSIM traffic simulator. It was demonstrated that the proposed method yielded better performance than several good-performance algorithms such as SAT [98].

Chu et al. [71] proposed a cooperative multi-agent framework to control a multi-intersection traffic signal system. It is based on the idea of independent Q-learning (IQL), i.e., multiple local RL agents are utilized to control each intersection in the system. However, instead of Q-learning, the authors used an advantage actor–critic (A2C) method, owing to its better performance than Q-learning methods, called multi-agent advantage actor–critic (MA2C). It improves upon the independent A2C methods by:

(i) Allowing each local agent to learn some features of the nearby agents to gain deeper localized traffic information;
(ii) Introducing a spatial discount factor in the reward function that weakens information transmission from the farther agents. The proposed method was tested on a simulated multi-intersection traffic scenario developed using SUMO and has been demonstrated to outperform SOTA algorithms.

Wang et al. [72] proposed a graph neural network-based framework for developing an intelligent multi-intersection traffic signal control system. It utilizes an adjacency graph to model the cooperation mechanism between the traffic lights, an RNN for incorporating historical traffic information with current traffic status, and a GNN to represent the relationship among the traffic lights. The proposed model was tested on both synthetic and real-world data and was shown to perform better than the SOTA.

Wang et al. [73] proposed a cooperative group-based adaptive traffic signal control framework. It used a nearest neighbor-based state representation and spatial factor-based discounted joint reward to improve the coordination among the traffic lights. It is one of the first approaches to combine reinforcement learning with a pheromone-based regional green-wave control mode that balances road congestion with road capacity. The proposed framework surpasses the cooperative group-based IQL algorithm in every metric evaluated in their work.

Lin et al. [74] tackled the problem of large-scale fleet management for ride-sharing platforms. The authors developed two novel MARL algorithms, contextual DQN and contextual actor–critic, that utilize geographical context information to reduce the action space complexity by eliminating the invalid action space, thereby reducing the computational cost incurred during agent training. In addition, they also developed a simulator to simulate traffic based on the real-world dataset collected by Didi Chuxing. The data consist of the number of hire orders and trajectories of each vehicle in the fleet collected in Chengdu, China. They demonstrated the effectiveness of the proposed algorithms under various experimental settings.

ITM has two main applications: automated intersection management (i.e., controlling traffic in the absence of traffic lights) and cooperative traffic signal control (i.e., controlling the traffic signal to reduce congestion). Both problems are generally classified as scheduling problems, and these can be easily modeled and solved using MARL. As discussed above, researchers utilized multi-agent variants of algorithms based on DQN [65,69,70] or actor–critic [71,74] to optimally schedule each traffic signal or CAV. DQN-based algorithms are suitable for traffic signal control since the action space of a traffic signal is discrete. Some researchers have employed RNNs [72] to remember historical information for better modeling. LSTMs can play a better role in modeling the sequential relation among nearby traffic signals. 

### 3.3. Sim-to-Real Approaches

In practical applications, agents might not know the actual model completely, including all agents’ reward functions and the transition probability model. This can create a disparity between an agent’s performance in a simulation and its performance in the real world, referred to as the sim-to-real gap. Tang et al. [75] proposed a motion prediction model using LSTM, and a deep ensemble technique was used to quantify epistemic and aleatoric uncertainty. The estimated uncertainty was incorporated into a decision-making framework using a potential field and model predictive control (MPC) [99] algorithm to reduce driving risk. The proposed uncertainty-aware decision-making framework was tested on various traffic scenarios from NGSIM and INTERACTION datasets and was also tested in a real-world scenario through a hardware-in-the-loop experiment bench.

Zhang et al. [76] proposed a MARL framework with a modified exploration strategy to solve the optimal parking assignment (OPA) problem and reduce the total travel time of connected vehicles (CVs) and non-connected vehicles (NCVs) by observing and dispatching CVs to appropriate parking lots. This MARL framework effectively allocates parking resources under different CV penetration rates, as demonstrated through simulations and comparisons with other baseline methods. The proposed model was tested in a simulated environment based on a real scenario within the campus of Tongji University.

Transferring technology from simulated environments to real-world environments is one of the most important aspects of the research work on MARL for CAVs. We have noted above that researchers attempted sim-to-real transfer for different tasks such as optimal parking assignment [75,76], real-time trajectory prediction, etc. It is important to note that the current research has mainly concentrated on controlled environments with lower traffic volume, slower traffic flow, and easily predictable vehicle trajectories based on their historical data. For an open-world environment, researchers may utilize domain adaptation techniques such as meta-reinforcement learning (meta-RL) or skill discovery to help generalize the model to previously unseen scenarios.

### 3.4. Safety

Most of the current multi-agent reinforcement learning (MARL) methods primarily focus on improving the performance of policies based on returns, but none guarantee safety during the learning process. However, it is essential to have MARL methods that can provide safety guarantees in safety-critical applications, such as robotics and autonomous vehicles, where exploration may lead to dangerous situations and potentially catastrophic outcomes if the agent malfunctions during deployment. 

ElSayed-Aly et al. [80] proposed a centralized shielding approach for MARL. This approach involves the creation of a single shield to monitor the joint actions of all agents and ensure their compliance with a safety specification. They have presented a new application of the max-plus algorithm for traffic control in lane-free environments. Additionally, they have also built upon the existing Flow framework and modified it to support connected vehicles in lane-free traffic simulation environments using SUMO.

Zhang et al. [77] developed an approach to predict the collision probability for safer motion planning. It consists of a risk-sensitive algorithm based on soft actor–critic [100] integrated with Lyapunov functions [101] in a model-free framework to increase system safety. They demonstrated the performance of their approach in a simulated scenario of autonomous navigation tasks. The following theorem serves as the basis for guaranteeing stability: under a given safety constraint, $d^{\prime }$, if there exists a function $L\left(s\right):S\to R_{+}$, and positive constants $\alpha_{1},\alpha_{2},\alpha_{3},\eta ,\;\mathrm{and}\;e$ such that:

(9) $$\eta <d^{\prime }$$

(10) $$e-\alpha_{1}\eta \leq \alpha_{3}d^{\prime }$$

(11) $$E_{t_{0}\sim \;P\left(t_{0}|\rho ,\pi \right),\;P\left(s|\pi ,\rho ,t_{0}\right)}\left[L\left(s\right)\right]<e$$

(12) $$\alpha_{1}c_{\pi }\left(s\right)\leq L\left(s\right)\leq \;\alpha_{2}c_{\pi }\left(s\right)\;\;\;\forall \;s\in S$$

and

(13) $$E_{s\sim \mu \left(s\right)}\left[E_{s^{\prime }\sim P_{\pi }}\left[L\left(s^{\prime }\right)-L\left(s\right)\right]\right]\leq -\alpha_{3}E_{s\sim \mu \left(s\right)}\left[c_{\pi }\left(s\right)\right]$$

where $\mu \left(s\right)=\left(\frac{1}{N}\right)\sum_{T=0}^{N}E_{t_{0}\sim P\left(t_{0}|\rho ,\pi \right)}P\left(s|\pi ,\rho ,t_{0}+T\right),\;s\in \Delta$ is the mean probability of being in s during a time in the edge set Δ under policy π.

Zhu et al. [78] attempted to create automated driving control models that can prevent safety hazards caused by distracted pedestrians with different crossing speeds at unsignalized mid-block crosswalks. They developed an original rule-based model that assumes all detected pedestrians are distracted and an original learning-based model using reinforcement learning methods with a comprehensive reward function that considers failure cases and acceptable minimum safety margin time for pedestrians. Pedestrian crossing action is modeled in (14) and (15).

(14) $$D_{AV}\left(t\right)\geq \left(T_{cross}^{\prime }+\beta \right)\times v_{AV}\left(t\right)\;$$

(15) $$T_{cross}^{\prime }=T_{cross}\times \left(1+\epsilon \right)$$

where $D_{AV}\left(t\right)$ is the distance between vehicle and pedestrian at time t. $T_{cross}^{\prime }$ is the expected leaving time from the conflict zone. $v_{AV}\left(t\right)$ is the speed of the vehicle at time t. $T_{cross}$ is the actual leaving time from the conflict zone. ϵ is the pedestrian’s estimation error on crossing time.

A hybrid approach combining rule-based and learning-based models is also proposed. The study compares the rule-based, learning-based, and hybrid models under a simulated scenario of unsignalized crosswalks developed using SUMO.

Bautista-Montesano et al. [79] presented a coupled RL and model predictive control (MPC) architecture for AV navigation, with both systems able to control the vehicle’s lateral and longitudinal dynamics independently. The RL and MPC algorithms run in parallel, and the output is selected based on a safety-oriented discrete selector. A T-intersection environment having a main road and a side road, with traffic following right-hand rules, was analyzed. 

The implementation of CAVs in the real world widely requires safety assurance. Two of the primary approaches to dealing with safety in CAVs are:

(i) Uncertainty quantification;
(ii) Lyapunov functions.

There are broadly two categories of uncertainties, epistemic and aleatoric. Aleatoric uncertainty is generated due to the inherent randomness/noise in the collected data. Thus, such an uncertainty is typically difficult or impossible to eliminate. On the other hand, epistemic uncertainty results from the lack of knowledge about the model. Therefore, applying various techniques, such as artificial potential fields, safety force fields, etc., can reduce epistemic uncertainty by quantifying uncertainty. Lyapunov functions [101] are scalar functions that can be used to guarantee the stability of a system. Safety can be modeled as a constraint in an MDP; therefore, the Lyapunov function to restrict the agent’s exploration ability within a boundary can be used to ensure “safe exploration” to prevent catastrophic actions such as collision or going out of the lane.

CAVs have the potential to revolutionize transportation and make it safer, more efficient, and more environmentally friendly. However, a variety of safety issues needed to be addressed in the implementation of CAVs. Here are some of the critical safety issues related to CAVs:

- Cybersecurity: CAVs are connected to the internet, which means they are vulnerable to cyberattacks. A cyberattack on a CAV could compromise the safety of passengers and other road users.
- Malfunctions: CAVs operate with complex sensors, software, and hardware. A malfunction in any of these components could lead to an accident.
- Personal data privacy: CAVS collect a vast amount of data about their passengers and surroundings. There is a risk that these personal data could be misused or hacked, compromising the passengers’ privacy.
- Legal liability: In the event of an accident involving a CAV, it may be challenging to determine who is legally liable. Is it the manufacturer of the vehicle, the software developer, or the vehicle’s owner? This also leads to a moral dilemma.
- Infrastructure compatibility: CAVs require a new, more sophisticated infrastructure to operate safely. The infrastructure must be compatible with the vehicles’ sensors and communication systems, which could be a significant challenge.
- Safety of humans and animals on the road: Ensuring the safety of pedestrians, cyclists, and other vulnerable road users such as animals is critical, and AVs must be designed to detect and respond to them. However, this can be particularly challenging in crowded urban environments.

These safety issues need to be addressed before CAVs can become a mainstream mode of transportation. Governments, manufacturers, and other stakeholders must work together to develop regulations and standards that ensure the safety of CAVs and their passengers.

### 3.5. Benchmarking Platforms

Benchmarking platforms serve an essential purpose to help validate the performance of a newly developed algorithm against the existing SOTA algorithms. Several past datasets have been created to evaluate the effectiveness of MARL algorithms for CAVs.

The development, testing, and benchmarking of behavior models frequently involve simulation, yet these simulations frequently employ datasets and behavior models that cannot fully capture the spectrum of interactive, real-world human behaviors. To this end, Bernhard et al. [81] introduced BARK, an open-source behavior benchmarking environment that addresses these issues by allowing behavior models to be reused for motion planning, prediction, and simulation. BARK includes a range of behavior models, such as Monte Carlo tree search [102] and reinforcement learning-based models. It was evaluated using a public dataset and utilizes a sampling-based scenario generation for velocity control of the ego-vehicle to demonstrate the inter-exchangeability of behavior models and how well they cope with interactions and changes in the model.

Yan et al. [82] presented a unified methodology for designing multi-agent vehicular systems and developing a corresponding CACC system using SOTA DRL algorithms. The performant behaviors discovered through the DRL method were manually analyzed, and simple controllers inspired by these behaviors were benchmarked in various road traffic scenarios such as single ring, double ring, figure of eight, highway ramp, and intersections. All the simulations were performed using the Flow framework in the SUMO simulator.

Palanisamy [83] has tackled the problem of connected and autonomous driving (CAD) to reflect real-world assumptions. This proposal suggests using partially observable Markov games to represent connected autonomous driving problems to reflect real-world assumptions. They also propose MACAD-Gym, a platform for learning with multiple agents that includes a range of simulation environments for studying CAD. These environments can be used to develop and evaluate DRL algorithms for various components of CAD systems, including sensing, perception, planning, and control. The simulations allow for the study of CAD systems in a variety of realistic, multi-agent settings with no limitations on the operational design domain.

Diehl et al. [84] proposed a model-based offline RL framework for safe trajectory planning and control of autonomous vehicles. It consists of a conditioned variational autoencoder (CVAE) to learn a stochastic forward dynamic model capable of estimating the actions of other vehicles based on the actions of the self-driving vehicle. In addition, it further incorporates the aleatoric uncertainty of the environment to improve the performance of planning-based approaches and has been established as a benchmarking platform. Table 2 provides the scopes, features, etc., in the benchmarking platforms discussed in this survey.

### 3.6. Datasets Widely Used in CAV Applications

CAV applications demand various datasets incorporating different scenarios to perform experimentations and research. Several researchers recommend and use a few popular publicly available datasets. The following are the brief descriptions of three popular publicly available datasets (useful in CAV applications):

- INTERACTION [42]: This dataset comprises realistic movements of different traffic participants in diverse, highly interactive driving scenarios across multiple countries. Further details and data formats are available on the dataset’s website. This dataset can facilitate research in several areas related to behavior, such as predicting intentions/motions/behaviors, cloning behaviors through imitation and inverse reinforcement learning, modeling and analyzing behavior, reinforcement learning, developing and verifying decision-making and planning algorithms, extracting and categorizing interactive behaviors, and generating driving scenarios/cases/behaviors.
- TrajNet++ [94]: A large-scale trajectory prediction benchmark dataset designed to evaluate the performance of trajectory prediction models. It contains over 78,000 pedestrian trajectories in various real-world scenarios. The dataset also includes high-resolution top-view images of the scenes and additional information such as pedestrian attributes (e.g., age, gender, clothing) and social groups. The TrajNet++ dataset provides evaluation metrics for trajectory prediction models, including average displacement error (ADE), final displacement error (FDE), and trajectory intersection over union (IOU). It also includes a baseline model and a leaderboard to facilitate fair comparison and benchmarking of different models.
- Next Generation Simulation (NGSIM) [95]: This is a collection of detailed traffic trajectory datasets from real-world traffic observations. It was collected by the US Federal Highway Administration (FHWA) at six different locations in the United States between 2005 and 2007. The NGSIM dataset includes vehicle trajectory data from video cameras and roadside sensors. The dataset contains individual vehicles’ position, speed, and acceleration as they move through the traffic network, as well as other characteristics such as vehicle type, length, and width.

## 4. Discussion

In this work, we have surveyed a range of papers covering various aspects needed for developing autonomous vehicles using the MARL approach. We have identified various trends and challenges in the current research.

- Spatiotemporal Data Analysis: Sequential data such as spatiotemporal traffic data containing information such as vehicles’ location, speed, inter-vehicle distance, etc., play a key role in solving CAV domain problems. To represent the relations and extract spatiotemporal features in the traffic data, graph-based networks such as graph convolution networks (GCNs) [103], graph attention networks (GATs) [104], etc., are used as they are capable of generating node embeddings which allow storing each object’s features as well as inter-related features [48,58,92]. They are used to solve various CAV problems such as vehicle platooning, lane merging, highway on-ramp merging, trajectory prediction, unsignalized intersection management, solving traffic congestion, and controlling traffic lights. Despite the current developments, there is a need to incorporate multi-modal spatiotemporal data to extract richer information that can help improve the performance of GCNs. Such data can be meteorological data on air quality, weather conditions, temperature, etc., to allow for more efficient traffic prediction vision-based data of the surroundings to provide contextual information during motion planning. Similarly, researchers can explore other CAV-related data to develop models for better decision making in CAVs.
- Domain Adaptation: Transferring the knowledge learned in a simulated environment to a real-world environment is called domain adaptation. This goal is often difficult to achieve in the CAV domain because the traffic environment data received by the sensors in the real world could belong to vastly different distributions compared to the training data seen in simulated environments. Another important related issue is inference time in the real-world environment. Various works have considered the sim-to-real transfer approach to tackle this challenge [75,77]. However, they only focus on localized CAV tasks such as optimal parking assignment to find the optimal parking spots for the autonomous vehicles in real time or performing navigation in a controlled environment such as indoor areas. In recent years, tremendous success has been achieved by meta-reinforcement learning (meta-RL) [105] techniques to perform zero-shot sim-to-real domain adaptation in robotics [105,106,107]. Hence, meta-RL-based approaches could be explored for their application to the CAV domain for efficient sim-to-real transfer. Nevertheless, another potential research topic in this area can be active learning [108], where the agents can consult human experts to improve the model’s decision-making ability in real time.
- Safety and Interpretability: Safety is one of the most critical factors for the real-time implementation of CAVs. CAVs are deployed in the real world where various actors, such as other vehicles (both CAVs and HDVs), pedestrians, and other entities, may co-exist and move independently. In such a scenario, CAVs must navigate safely and comfortably by avoiding possible collisions. Several works have utilized constraint satisfaction methods by defining the Lyapunov function for the vehicle’s behavior policy, defining safety specifications, and regulating the vehicle’s actions through shielding to avoid “unsafe” actions. Nonetheless, there exists a need for model interpretability [109] to provide safety guarantees for the real-world deployment of CAV systems. Hence, researchers can also explore the possibility of developing an interpretable MARL model for CAVs.
- Benchmarking Platforms and Datasets: Developing benchmarking platforms and curating datasets play a crucial role in validating the usability of MARL algorithms. The CAV domain spans a variety of tasks, such as cooperative motion planning, trajectory prediction of CAVs and pedestrians, automated traffic light control, and automated traffic management [79]. Some researchers have developed and proposed benchmarks for behavior models for a limited number of tasks such as controlling ego-vehicle velocity in a multi-agent environment. Hence, there is a need to develop more advanced benchmarking platforms to cover a more extensive range of CAV tasks.
- Communication Issues: Beyond the algorithmic and data-based research, communication among the CAV agents in an environment heavily influences the performance of CAV systems. Information transmission delay in a CAV network can become catastrophic and life-threatening in a real-world scenario. It is another open challenge for researchers to consider information transmission delay in the CAV network while developing MARL algorithms [19].

Apart from several developments in CAVs, there are several reasons behind the slower market transition from normal vehicles to full CAVs. A few of them are as follows:

- Technical Challenges: Developing full AVs is a complex and cumbersome process that requires advanced hardware and software systems, such as sensors, ML algorithms, and AI. These technologies are still evolving, and technical challenges need to be overcome, such as ensuring the safety and reliability of the system.
- Consumer Acceptance: Consumers are reluctant to adopt CAVs due to concerns about safety, reliability, and loss of control. Education and awareness campaigns are necessary to help consumers understand the benefits of CAVs and overcome their concerns.
- Infrastructure: The deployment of CAVs requires a supportive infrastructure, such as ITSs, advanced communication networks, and charging stations for electric vehicles. The lack of infrastructure also hinders the deployment and adoption of CAVs.
- Cost: The development and deployment of CAVs require significant investment, and the cost of the technology is still relatively high. Therefore, many consumers and businesses cannot afford the high cost of CAVs, which can limit their adoption.
- Regulatory and Legal Issues: The deployment of CAVs raises a number of legal and regulatory issues, including liability, data privacy, and cybersecurity. Governments and regulators need to establish clear rules and regulations to ensure the safety and security of the public when using these vehicles.

Therefore, the transition to fully connected and automated vehicles will require collaboration between the private and public sectors to overcome these challenges and create a supportive ecosystem for the widespread adoption of CAVs.

## 5. Conclusions

In this survey, we have detailed the ongoing research works in the MARL application field to the CAV domain. These works were classified under various categories to highlight the current approaches, and their corresponding mechanisms were explained to provide a brief idea of each work. We also discussed various simulators actively used to design the CAV scenarios and test the proposed MARL algorithms. In addition, several challenges are discussed to underscore the representative problems in the SOTA and propose potential directions to tackle those problems. The uncertainty of the real world requires such deployment MARL for CAVs. We have categorized different tasks of the CAV that require multi-agent settings to divide the challenge among different agents and conquer the seen and unseen situations. However, testing the model in a simulated environment is recommended before deploying it in the real world. It helps adapt a new domain and removes the burden of database generation for plenty of tasks for real-world deployment. It also reduces the cumbersome and costlier process of manual data curation.

Further, by considering competitive and cooperative agents in the MARL settings, performance of the model is enhanced. Therefore, we also recommend involving both competition and collaboration in the learning policies of the agents. Furthermore, human in the loop (HITL) improves prediction performance in an uncertain environment. Therefore, HITL-based continual learning during the model’s deployment expands the model’s domain and prepares the model for the open-world novelty.

## Figures and Tables

**Table 2:** Key features and scope of the benchmarking platforms surveyed.

Reference No.	Key Features	Scope	Flexibility	Accessibility
Bernhard et al. [81]	Systematic evaluation and improvement of vehicle behavior models.	Vehicle behavior models.	Capable of extending to future behavior models beyond the original reference implementations.	Open source.
Yan et al. [82]	Provides unified multi-agent, multi-task reinforcement learning methodologies to simulate vehicular systems in mixed autonomy traffic.	Various deep reinforcement learning algorithm implementations for decision-making tasks in AVs.	Capable of extending to more complex traffic scenarios.	Open source.
Palanisamy [83]	Provides a multi-agent autonomous driving platform for simulating various kinds of driving environments and diverse types of agents.	Various driving environments with diverse driving agents.	Capable of introducing more complex types of driving environments depending on user’s need.	Open source.
Diehl et al. [84]	Provides an uncertainty-aware model-based offline planning framework for tackling uncertainties in real-world driving scenarios.	Uncertainty-aware autonomous driving framework.	Difficult to extend the existing capabilities due to the complex implementation.	Open source.

## Data Availability

The data used to support the findings of this study are included in this article.
